# Signet Ring Cell Carcinoma of the Lung: A Diagnostic Pitfall in Pregnancy

**DOI:** 10.1155/2019/9461579

**Published:** 2019-06-12

**Authors:** Sabine Danzinger, Wolfgang J. Köstler, Martin Funovics, Merima Herac, Leonhard Müllauer, Helmut Prosch, Heinz Kölbl

**Affiliations:** ^1^Department of Obstetrics and Gynecology, Comprehensive Cancer Center, Medical University of Vienna, 1090 Vienna, Austria; ^2^Clinical Division of Oncology, Department of Medicine I, Comprehensive Cancer Center, Medical University of Vienna, 1090 Vienna, Austria; ^3^Department of Biomedical Imaging and Image-Guided Therapy, Comprehensive Cancer Center, Medical University of Vienna, 1090 Vienna, Austria; ^4^Department of Pathology, Comprehensive Cancer Center, Medical University of Vienna, 1090 Vienna, Austria

## Abstract

Lung cancer during pregnancy represents a rare disease. In this case report, we present a patient at advanced and metastasized stage of signet ring cell carcinoma who presented in the 22^nd^ week of gestation.

## 1. Introduction

Lung cancer during pregnancy is very rare and often detected at an advanced stage with poor prognosis [1–4]. In the past decades, pregnancy associated lung cancer has become more common due to arising trends of cigarette smoking among young women, delaying childbearing, and increased incidence of lung cancer worldwide [5–7]. Whereas most lung cancers are adenocarcinomas, primary signet ring cell (adeno)carcinoma (SRCC) of the lung represents a rare uncommon condition, originally described by Kish et al. in 1989 [8]. The incidence varies from 0.14% to 1.9% of all lung cancers [9].

The purpose of this case report is to describe a primary SRCC during pregnancy, representing an extremely rare disease.

## 2. Case Presentation

**Figure 1:**
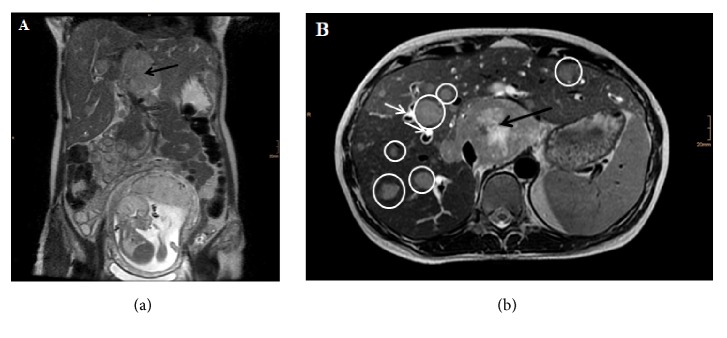
Magnetic resonance imaging (MRI) shows an enlarged liver with central tumor (black arrow), dilated biliary ducts (white arrows), and disseminated metastases (circles) of a pregnant woman at 20 weeks 4 days of gestation. (a) coronal, (b) axial view.

A 37-year-old woman, gravida 2, para 0, with a suspicious tumor at the liver hilum at 21 weeks 5 days of gestation was admitted to the department of obstetrics and gynecology of our hospital. Physical examination revealed a very sick and suffering patient. The patient presented with dyspnoea, jaundice, epigastric pain, ascites, and abdominal tenderness. Orange urine and white stool were reported. There was no previous or family history of any cancer. No regular medication was reported. The patient was a former light smoker; she did not smoke during pregnancy. Magnetic resonance imaging (MRI) had been performed eight days before the patient's admission; it revealed an enlarged liver with a centrally located tumor at the liver hilum and disseminated hepatic and abdominal lymph node metastases. MRI also displayed mechanical cholestasis with dilated biliary ducts and ascites (Figure 1).

On obstetrical ultrasonography at the time of admission, the estimated fetal weight was 470 g (59^th^ percentile), a Doppler measurement of blood flow through the uterine arteries was performed, and the median PI (pulsatile index) was below 1.5. Fetal movements, fetal anatomy, placenta, amniotic-fluid volume, and the length of the cervix (40.0 mm) were normal.

At the time of the patient's initial presentation, laboratory results showed severe normocytic normochromic anemia with haemoglobin level 6.7 g/dl and hematocrit 20.2%, leukocytosis with a white blood cell count of 14.47 G/l, and a normal platelet count. Liver function parameters were elevated as follows: total bilirubin 16.64 mg/dl, glutamate oxaloacetate transaminase (GOT) 70 U/l, glutamate pyruvate transaminase (GPT) 42 U/l, and gamma-glutamyl transferase (GGT) 90 U/l. Cholinesterase was <1 kU/l; both alkaline phosphatase (843 U/l) and lactate dehydrogenase (LDH) (635 U/l) were elevated. Total protein and albumin levels were reduced (52.5 g/l, 28.0 g/l, respectively). Blood coagulation analysis resulted in a prothrombin time of 34%; an activated partial thromboplastin time (APTT) was 45.1 s. Fibrinogen (279 mg/dl) was normal. A high level of C-reactive protein (CRP) (8.23 mg/dl) was detected. Common kidney function parameters and serum electrolytes were normal. These laboratory findings are shown in Table 1. Tests for viral hepatitis B and hepatitis C and HIV were all negative.

**Figure 2:**
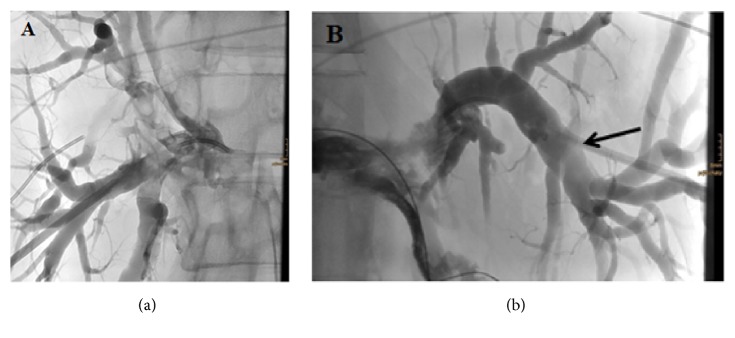
Percutaneous transhepatic biliary drainage (PTBD): consecutive puncture of the right and left biliary ducts, separated by the tumor; catheter in the dilated left biliary ducts (arrow). (a) right (b) left side.

**Figure 3:**
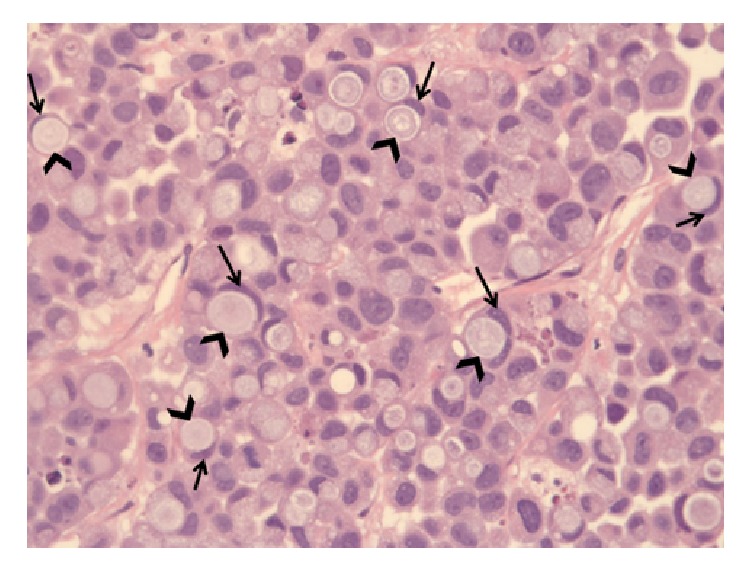
The histopathological examination of the liver-biopsy specimens showed a poorly differentiated, diffusely infiltrating signet ring cell carcinoma (SRCC), grade 3. SRCC consists of signet ring cells, containing abundant intracytoplasmic mucin (arrowheads) pushing the nucleus (arrows) to the periphery. Hematoxylin-eosin stain, x40 magnification.

**Figure 4:**
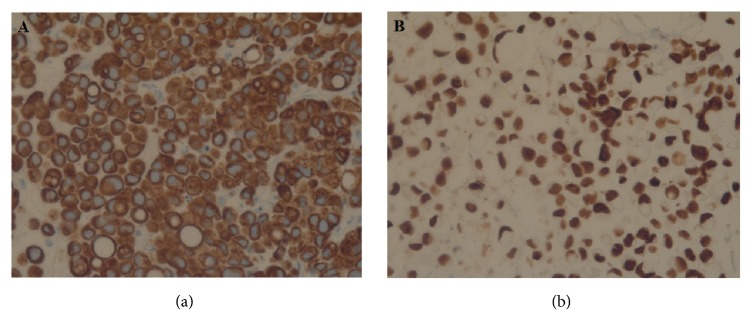
Immunohistochemistry revealing tumor cells positive for cytokeratin (CK) 7 (a) and thyroid transcription factor-1 (TTF1) (b). Immunostaining, x40 magnification.

Acute hepatic failure was diagnosed. According to the findings, the patient underwent a percutaneous transhepatic biliary drainage (PTBD) (Figure 2). Pathological examination of the liver-biopsy specimens, obtained at PTBD, revealed a poorly differentiated, diffusely infiltrating SRCC, grade 3 (Figure 3). By immunohistochemistry, the tumor was positive for cytokeratin (CK) 7 (Figure 4(a)). Tumor cells were negative for CK20, caudal-type homeobox transcription factor 2 (CDX2), estrogen receptor (ER), progesterone receptor (PR), PAX8, and human epidermal growth factor receptor 2 (HER2). Positive expression of programmed death-ligand 1 (PD-L1) was found in 30% of the tumor cells. Immunohistochemistry of phosphatase and tensin homolog (PTEN) was weak and not conclusive. In conclusion, histomorphology and immunohistochemical findings argued for a primary tumor of the upper gastrointestinal tract. DNA repair proteins like MLH1, MSH2, MSH6, and PMS2 were positively expressed. Thus, these findings argued against microsatellite instability. Next generation sequencing-based analysis of common targetable cancer mutations was ordered. The Ion AmpliSeq™ Cancer Hotspot Panel v2 (Ion Torrent™) and the Ion AmpliSeq™ BRCA1 and BRCA2 Panel (Thermo Fisher Scientific Inc., Waltham, MA, USA) were used for mutational analyses.

The following day, recurrence of ascites was observed. Therefore ultrasound-guided paracentesis was performed.

Two days after admission, regular labour started spontaneously at 6 am; therefore the patient was transferred to the delivery room. Immediately, the patient had rupture of membranes and delivered a female infant in pelvic presentation, by spontaneous vaginal delivery, at full 22 weeks of gestation. The baby weighed 427 g; the Apgar scores at 1, 5, and 10 minutes were all 1. The placenta seemed cleft; thus ultrasonography of the uterine cavum was performed, and no placental residues were detected. In general, loss of blood and involution of uterus were normal.

**Figure 5:**
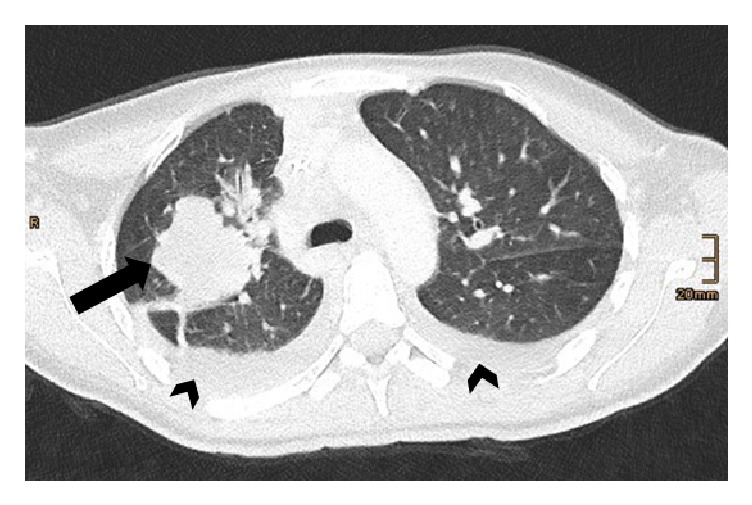
Chest computed tomography (CT) scan demonstrates a mass lesion (5 cm) in the right upper lobe of lung (arrow), and bilateral pleural effusions (arrowheads).

Afterwards, an abdominal CT (computed tomography) scan detected metastatic disease, a large mass located at the liver hilum (6 cm at maximum) next to the gastric cardia, hepatic metastasis, cholangiectasis, and enlarged locoregional lymph nodes, and bone metastases in the whole axial skeletal system. Additionally, the CT showed signs of a paralytic ileus; therefore a stomach tube has been inserted. A chest CT scan demonstrated a large right upper lobe mass (5 cm in diameter), appearing malignant, and bilateral pleural effusions (Figure 5).

In conjecture, histopathologic and imaging findings were most consistent with a primary signet cell carcinoma of gastric origin.

Despite antibiotic coverage with ampicillin after PTBD, the patient developed clinical signs of sepsis including tachycardia, fever, dyspnoea, and low blood pressure, along with significantly elevated procalcitonin (PCT) levels (5.12 ng/ml). PCT is used as a biomarker for the diagnosis of sepsis, and PCT is used to guide antibiotic therapy [10]. Initially, no specific focus of sepsis was found. Accordingly, antibiotic therapy was changed by replacing ampicillin with piperacillin/tazobactam.

Blood cultures later grew Candida albicans and streptococci. Teicoplanin and fluconazole were thus added to the piperacillin/tazobactam regimen. Moreover, polymerase chain reaction (PCR) for DNA quantification of CMV revealed 1.63 x 10^2^ c/ml. Thus, valganciclovir was administered.

Seven days after admission, an ultrasound examination of the liver demonstrated minimal intrahepatic cholangiectasis and gallbladder wall thickening. Decompression effect and clinical improvement were observed, but there was no improvement in laboratory parameters of cholestasis with bilirubin levels plateauing at 26.87 mg/dl.

Antitumor therapy was discussed: Because of her poor performance status (ECOG 3) and cholestasis, the patient was not a candidate for chemotherapy, and beyond PD-L1, no drugable targets were discovered in immunohistochemical analyses. After careful discussion with the patient and her family and after obtaining informed consent experimental therapy with pembrolizumab (200 mg as intravenous infusion) was initiated and well tolerated. Pembrolizumab represents a monoclonal antibody which binds to PD-1, blocking the interaction between PD-1 and its ligands (e. g. PD-L1) [11, 12]. In tumor tissues, binding of PD-1 on T-cells to PD-L1 expressed by tumor cells inhibits the antitumor immune response of T-cells, thus enabling immune escape of tumor cells and neoplastic growth [11, 13, 14].

A few days later, the results from sequencing analyses were available: Sequencing results revealed a deletion in exon 19 of the epidermal growth factor receptor (EGFR) [p.(E746_A750del)], which represents a prototypical mutation characterizing as subset of adenocarcinomas of the lung. In addition, mutations of RB1 (exon 22) and TP53 (exon4) were detected. The patient did not have a BRCA1/2 mutation. Additional immunohistochemical stains ordered based on sequencing results revealed that the tumor cells were highly positive for thyroid transcription factor-1 (TTF1), which represents a useful marker in the diagnosis of tumors of thyroid or lung origin (Figure 4(b)) [15, 16].

Based on the mutational profile, treatment with afatinib was recommended. Afatinib shuts down the signalling activity of receptor tyrosine kinases of the ErbB family by binding to three members of the family, namely, EGFR, HER2 and HER4, and by inhibiting the transphorylation of a fourth member, HER3 [17]. Owing to her rapidly deteriorating health status, the patient was not able to commence afatinib. Despite supportive measures the patient died on the 14^th^ day of admission.

Taken together, this pregnant woman was diagnosed with signet ring cell carcinoma which was primary assumed to originate from the upper gastrointestinal tract. In addition, liver and bone metastasis and a malignant-appearing lung lesion were observed. According to the histopathological (CK7 positive, CK20 negative, TTF1 positive) and genetic findings (mutation of EGFR, exon 19), primary SRCC of the lung was the final diagnosis.

## 3. Discussion

Cancer during pregnancy is a rare occurrence with an estimated incidence of 1 in 1000 pregnancies. Breast and cervical cancer, malignant melanoma, and haematological malignancies are the most frequently diagnosed malignant diseases during pregnancy [18–23]. As there has been a rising trend of delayed childbearing over the last decades and the incidence of malignant diseases is known to be higher with advancing age, the incidence of cancer diagnosed during pregnancy has increased [19, 20, 22, 24].

We present a case of signet ring cell carcinoma which was diagnosed in the 22^nd^ week of gestation. According to the initial imaging and initial histopathological findings, the primary tumor was assumed to originate from the upper gastrointestinal tract. However, the findings in a chest CT scan (tumor mass in the upper lobe with appearance of a primary pulmonary carcinoma) in combination with the results from immunohistochemistry (positive CK7 and negative staining for CK20, positive TTF1), and genetic analysis (mutation of EGFR), argued for primary lung cancer.

Lung cancer is estimated to account for 12% of all new cancer diagnoses in women. The probability of developing lung cancer is 0.2% (1 in 598) under the age of 49 [25]. Lung cancer in pregnancy is a very rare situation. Due to arising trends of cigarette smoking among young women, delaying childbearing, and increased incidence of lung cancer worldwide, pregnancy associated lung cancer has become more common in the past decades [5–7].

Lung cancer during pregnancy is commonly diagnosed in advanced stage with poor prognosis [1–4]. There might be various reasons for a delay in diagnosis and poor prognosis. Lung cancer-associated symptoms, such as general fatigue, dyspnea, and cough, are often considered to be related to pregnancy. Furthermore, physicians hesitate to subject a pregnant patient to radiological assessment, due to radiation exposure [26]. In general, physicians are concerned about performing the diagnostic interventions required for cancer diagnosis in pregnant women [27].

In a review of Mitrou et al. in 2016, the authors reported on 66 published cases of gestational lung cancer. The median age was 36 years (17-45 years); the median gestational week was 27.3 weeks (8-38 weeks). 82% of the pregnancy associated lung cancer cases were non-small cell lung cancer (NSCLC) and 18% small cell lung cancer (SCLC). 35% of patients had a tobacco history, it was absent in 27%, and in 38% of patients there was no available information. At presentation, advanced stage of disease (III-IV) was diagnosed in 97% of women. Platinum-based chemotherapy was the most common treatment modality, resulting in no major responses. Six patients were treated with targeted therapies (erlotinib, n=2; gefitinib, n=1; erlotinib followed by gefitinib, n=1; crizotinib, n=2). All these patients were found to be positive for EGFR mutations or EML4 (echinoderm microtubule-associated protein-like 4)-ALK (anaplastic lymphoma kinase) translocations. Maternal survival was poor; 12% of patients died within one month postpartum. On the contrary, 12 patients (18%) were alive 12 months or more from diagnosis. These patients were diagnosed mainly with an early stage disease. 82% of the newborns were born healthy. Fetal and placental metastases were reported in 3 (4.5%) and 11 (17%) cases, respectively. Abortion was induced in 6 cases, and one spontaneous abortion was observed [2, 4–6, 26–30].

Boussios et al. reported on nine patients suffering from lung cancer during pregnancy. All patients presented with metastatic disease including bone, lung, brain, spinal cord, pleura, lymph nodes, adrenal, and liver [6]. The case of a pregnant woman with NSCLC and disseminated pulmonary and bone metastases and malignant pericardial and pleural effusions was described by Jackisch et al. This patient died within one month after diagnosis [1]. Ceauşu et al. presented a case of gestational lung adenocarcinoma with metastasis in the liver and ovaries [29]. In addition, involvement of the placenta and the fetus by tumor cells have been described [1, 4]. Metastasis to the ovary from SCLC during pregnancy was reported [31]. In the case of our patient, there were disseminated liver and bone metastases and bone marrow carcinosis.

Yates et al. presented the case of a pregnant woman who was diagnosed with stage IIIA poorly differentiated squamous cell carcinoma of the lung with lymphoepithelioma-like features at 18^th^ weeks of gestation. This patient received neoadjuvant chemotherapy with cisplatin and docetaxel, resulting in complete response. After delivery of a healthy baby at 35 weeks of gestation, she received radiation. The disease-free survival was more than 16 months after initial diagnosis [32].

In our case, the histopathological examination revealed a grade 3 SRCC. SRCC mostly occurs in the stomach, but also breast, colon, prostate, and the urinary bladder are rarely affected. Primary SRCC of the lung is a very rare disease, originally described by Kish et al. in 1989 [8]. The incidence varies from 0.14% to 1.9% of all lung cancers [9]. Tsuta et al. reported on 2640 surgically resected primary lung carcinomas; 39 (1.5%) of these tumors showed SRCC components. The authors showed a positive correlation between the size of the SRCC component of the tumor and the aggressiveness of the tumor and poor outcome [33]. In general, patients with SRCC tend to be nonsmokers compared to patients with other types of adenocarcinoma of the lung [34]. Iwasaki et al. analyzed 649 primary lung cancer cases; SRCC components (≥5%) were found in 7 tumors (1.1%). Patients with these tumors had a significantly worse overall survival (OS) compared to patients whose tumors had SRCC components of only <5%: median survival time 46.7 months (95% confidence interval (CI): 30.3, 63.1) versus 90.0 months (95% CI: 73.6, 106.3; p=0.0319), respectively [35]. 262 primary SRCC of the lung were compared to 50089 patients with lung adenocarcinoma by Ou et al. The authors demonstrated that the patients with SRCC were significantly younger and had a significantly higher proportion of poorly differentiated tumor and stage IV disease than patients with adenocarcinoma. Patients with SRCC had worse median OS (6 months) in comparison with other adenocarcinomas (10 months) (hazard ratio: 1.507; 95% CI: 1.326-1.714; p<0.0001). SRCC had similarity in clinicopathologic characteristics with EML4-ALK positive NSCLC [36]. Additionally, several reports on primary SRCC of the lung should be mentioned [9, 34, 37–41].

Our patient was suffering from a primary SRCC of the lung. Because of the rareness of the disease, it is important to distinguish between primary SRCC of the lung and metastatic one from other sites which are more common. Merchant and colleagues investigated 32 SRCC from various organs (17 lung, 5 breast, 5 stomach, and 5 colon) immunohistochemically. TTF1 was positively expressed in 14 (82.4%) pulmonary SRCC, but TTF1 was not expressed in any SRCC of the other organs. CK7 positivity and negative CK20 were shown in 94.1% of pulmonary SRCC [42]. The combination of positive TTF1 and CK7 expression and negative CK20 revealed a significant association with primary adenocarcinoma of the lung [43]. There are several studies and reports resulting in similar immunohistochemical findings [9, 15, 16, 34, 38, 39, 44–46]. In our case, these histopathological findings (CK7 positive, CK20 negative, TTF1 positive) confirmed the diagnosis of a primary SRCC of the lung.

Clinicopathological features, including the molecular genotype, of NSCLC diagnosed during pregnancy or the peripartum period, were described by Dagogo-Jack and colleagues. The authors performed a retrospective analysis of consecutive patients with NSCLC seen at their institution between 2009 and 2015. From the 2,422 women with NSCLC, 160 women of reproductive age, defined as 18 to 45 years old, were identified. Among these 160 women, eight (5%) were diagnosed with NSCLC during pregnancy/peripartum. All of these patients were minimal (n=2) or never-smokers (n=6) with metastatic adenocarcinoma. The median age at diagnosis was 35 years (29-43 years); the disease was diagnosed in all trimesters. ALK rearrangements were found in six patients; the remaining two were carriers of an EGRF mutation. All six patients with ALK translocations received the ALK inhibitor crizotinib; both women with EGFR mutation were treated with gefitinib or erlotinib, as first tyrosine kinase inhibitors (TKIs). None of the women were treated with targeted therapies during pregnancy; they received these agents after delivery. The authors conclude that genomic testing should be performed in patients with NSCLC diagnosed during pregnancy/peripartum to offer genotype-directed agents [47].

The use of erlotinib throughout pregnancy in a patient with stage IV lung adenocarcinoma with mediastinal, bone, and cerebral metastasis, a mutation of EGFR, and no smoking history was described by Rivas et al. After eight months of treatment, complete bone and central nervous system response and partial lung and mediastinal response were observed [48]. Furthermore, good response to treatment with EGFR TKI in a pregnant woman with metastatic lung adenocarcinoma was described [49]. A nonsmoking woman with twin pregnancy after in vitro fertilization was diagnosed with stage IV EGFR mutated NSCLC with cerebral metastases at 10 weeks of gestation. She was treated with stereotactic radiotherapy and erlotinib. At 33 weeks, intrauterine growth restriction (IUGR) was observed in one twin, leading to cesarean delivery at 37 weeks. Both twins had small weights (87% of expected). Partial response was shown 4 weeks postpartum. At 13 months postpartum, the patient received erlotinib and worked full-time; both twins were in good health [50].

Our patient received pembrolizumab, and afitinib was recommended. Gil et al. reported on therapy with gefitinib which was administered to a pregnant woman who presented with disseminated EGFR-mutated lung carcinoma with respiratory distress at 26 weeks of gestation. Targeted therapy resulted in rapid improvement of the respiratory symptoms allowing a planned Caesarean section on week 35, and giving birth to a healthy baby. The maternal progression-free survival was 42 weeks; the patient died 22 months after lung cancer was diagnosed [2].

In conclusion, we report on a pregnant woman with signet ring cell carcinoma of the lung, representing an extremely rare situation. Pregnancy-associated lung cancer is often detected at an advanced stage with poor prognosis. Therefore, early diagnosis and intervention are very important in case of lung cancer during pregnancy. Multidisciplinary management of this disease is essential for the patient's optimal treatment.

## Figures and Tables

**Table 1:** Laboratory results at the time of admission (MCV = mean corpuscular volume; MCH = mean corpuscular hemoglobin concentration; AST = aspartate aminotransferase; GOT = glutamate oxaloacetate transaminase; ALT = alanine aminotransferase; GPT = glutamate pyruvate transaminase; GGT = gamma-glutamyl transferase; LDH = lactate dehydrogenase; CRP = C-reactive protein; APTT = activated partial thromboplastin time).

	Trend	Result	Reference range	Unit
*Blood count*				
Red blood cell count	↓	*2.3*	3.8-5.2	T/l
Hemoglobin	↓	*6.7*	12.0-16.0	g/dl
Hematocrit	↓	*20.2*	35.0-47.0	%
MCV		89.4	78.0-98.0	fl
MCH		29.6	27.0-33.0	pg
White blood cell count	↑	*14.47*	4.0-10.0	G/l
Platelet count		217	150-350	G/l
*Clinical chemistry *				
Total bilirubin	↑	*16.64*	0.0-1.2	mg/dl
Total protein	↓	*52.5*	64-83	g/l
Albumin	↓	*28.0*	35-52	g/l
Cholinesterase	↓	*<1*	3.65-12.92	kll/l
Alkaline phosphatase	↑	*843*	35-105	U/l
AST (GOT)	↑	*70*	<35	U/l
ALT (GPT)	↑	*42*	<35	U/l
GGT	↑	*90*	<40	U/l
LDH	↑	*635*	<250	U/l
*Blood coagulation *				
Owren prothrombin time	↓	*34*	70-125	%
APTT	↑	*45.1*	27.0-41.0	s
Fibrinogen (Clauss)		279	200-400	mg/dl
*Immunoreaction*				
CRP	↑	*8.23*	<0.5	mg/dl

## Abbreviations

ALK: Anaplastic lymphoma kinase
ALT: Alanine aminotransferase
APTT: Activated partial thromboplastin time
AST: Aspartate aminotransferase
CDX2: Caudal-type homeobox transcription factor 2
CI: Confidence interval
CK: Cytokeratin
CMV: Cytomegalovirus
CRP: C-reactive protein
CT: Computed tomography
EGFR: Epidermal growth factor receptor
EML4: Echinoderm microtubule-associated protein-like 4
ER: Estrogen receptor
GGT: Gamma-glutamyl transferase
GOT: Glutamate oxaloacetate transaminase
GPT: Glutamate pyruvate transaminase
HER2: Human epidermal growth factor receptor 2
IUGR: Intrauterine growth restriction
LDH: Lactate dehydrogenase
MCH: Mean corpuscular hemoglobin concentration
MCV: Mean corpuscular volume
MRI: Magnetic resonance imaging
NSCLC: Non-small cell lung cancer
OS: Overall survival
PCR: Polymerase chain reaction
PCT: Procalcitonin
PD-L1: Programmed death-ligand 1
PI: Pulsatile index
PR: Progesterone receptor
PTBD: Percutaneous transhepatic biliary drainage
PTEN: Phosphatase and tensin homolog
SCLC: Small cell lung cancer
SRCC: Signet ring cell carcinoma
TKI: Tyrosine kinase inhibitor
TTF1: Thyroid transcription factor-1.
